# Phosphoproteomic Data Integration Reveals Paralog-Specific Co-Regulatory Networks of Rho-Associated Kinases ROCK1 and ROCK2

**DOI:** 10.3390/ijms27188121

**Published:** 2026-09-12

**Authors:** Chrysilla Espy Vaz, Manasa Suresh, Leona Dcunha, Rajesh Raju, Saptami Kanekar

**Affiliations:** Centre for Integrative Omics Data Science, Yenepoya (Deemed to Be University), Mangalore 575018, Karnataka, Indialeonajd.ciods@yenepoya.edu.in (L.D.)

**Keywords:** ROCK1, ROCK2, rho-associated coiled-coil containing protein kinases, phosphorylation, phosphosite conservation, signaling networks

## Abstract

Rho-associated coiled-coil containing protein kinases 1 and 2 (ROCK1 and ROCK2) are serine/threonine kinases in the AGC family that regulate cytoskeletal dynamics, cell morphology, and mechanosignaling. Although their primary cellular functions are well established, their phosphoregulatory mechanisms remain insufficiently characterized at the system level. In this study, the analysis of 3825 human cell line phosphoproteomic datasets identified Class I phosphosites (localization probability ≥ 75% or A-score > 13) in both ROCK1 and ROCK2. The predominant phosphosites were ranked according to their frequency across the datasets. Co-regulation with phosphosites in other proteins (PsOPs) was assessed using Fisher’s exact test with multi-criteria filtering. From 607 profiling and 153 differential datasets for ROCK1, and 667 profiling and 143 differential datasets for ROCK2, S1105 and S1341 for ROCK1, and S1137 and S1374 were considered the major ROCK2 phosphosites, all located outside the kinase domain. ROCK1_S1105 exhibited significant positive co-regulation with 141 PsOPs, whose annotated functions include cytoskeletal organization, cardiomyocyte survival, metabolism, and genomic stability, suggesting potential functional coordination. ROCK1_S1341 also displayed similar associations. In contrast, ROCK2_S1374 was associated with a broader network of 1349 PsOPs involved in cytoskeletal remodeling, cell cycle progression, hypertrophy, and fibrosis. Functional analyses identified both shared and paralog-specific regulatory networks, with C-terminal phosphosites as potential hubs associated with cytoskeletal regulation and the MAPK, PI3K/AKT/mTOR, and Wnt signaling pathways. ROCK2 exhibits co-regulation with a substantially larger network of phosphosites in proteins other than ROCK1, despite the positional conservation of the predominant sites, suggesting paralog-specific regulatory differences. This provides a hypothesis-generating resource for prioritizing ROCK phosphosites and co-regulated proteins as candidates for experimental validation in pathological contexts and for therapeutic target discovery.

## 1. Introduction

Rho-associated coiled-coil containing protein kinases (ROCK1 and ROCK2) are serine/threonine kinases belonging to the protein kinase A/G/C (AGC) superfamily, which function as key effectors of the small GTPase RhoA [1,2,3]. The mammalian Rho-associated protein kinase (ROCK) family consists of two paralogs, ROCK1 (also known as ROK *β* or p160ROCK) and ROCK2 (also known as ROK*α*) [4]. ROCK2 was discovered in 1995 through expression cloning and affinity purification as a novel ~150 kDa serine/threonine kinase that specifically binds to GTP-bound RhoA and translocates to peripheral membranes, establishing it as a direct downstream effector of RhoA signalling. ROCK1 was later identified as an isoform of ROCK2; however, according to the current nomenclature, ROCK1 and ROCK2 are more precisely classified as paralogs, being products of distinct genes [5,6]. The two mammalian paralogs share 65% overall amino acid sequence identity and 92% identity within their kinase domains, yet exhibit non-redundant tissue distributions and cellular functions [7]. ROCK2 also has a splice variant, ROCK2m, which is specifically expressed in the skeletal muscle and results in the inclusion of 57 additional amino acids [8]. ROCK2 is enriched in the brain and myocardium, whereas ROCK1 is more broadly expressed in the lung, liver, spleen, kidney, and testis [1,3,8,9]. Both paralogs are predominantly cytoplasmic and translocate to the plasma membrane after their activation.

Structurally, ROCK1 (1354 residues; chromosome 18q11.1) and ROCK2 (1388 residues; chromosome 2p24) share a conserved architecture comprising an N-terminal kinase domain, a central coiled-coil region harboring the Rho-binding domain (RBD), and a C-terminal pleckstrin homology (PH) domain that encompasses a cysteine-rich subdomain (CRD) [3,10,11]. An autoinhibitory mechanism operates through intramolecular interactions between the kinase domain and the C-terminal PH domain, which is relieved upon binding of GTP-loaded RhoA, RhoB, or RhoC to RBD [12,13]. N-terminal homodimerization further augments ROCK autophosphorylation and its catalytic activity [14]. In apoptotic contexts, caspase-3 cleaves ROCK1 to generate a constitutively active kinase fragment, whereas Granzyme B and caspase-2 activate ROCK2 [15,16]. In a non-apoptotic scenario, thrombin activates caspase-2 in endothelial cells and increases ROCK2 expression, resulting in increased caspase-2-mediated proteolytic cleavage, ROCK2 activation, and subsequent microparticle formation [17].

Protein phosphorylation is a fundamental mechanism for modulating kinase activity, influencing localization, and regulating protein–protein interactions. Although ROCK1 and ROCK2 have been widely characterized as kinases that phosphorylate substrates such as myosin light chain (MLC), LIMK, and MYPT1 which regulate actomyosin contractility and cytoskeletal tension [18,19,20], systematic analysis of ROCK1 and ROCK2 phosphorylation in the context of global phosphoproteomic datasets remains limited. Understanding the sites and frequency of ROCK1 and ROCK2 phosphorylation in diverse human cellular contexts is critical for interpreting paralog-specific signaling and identifying regulatory nodes that respond to therapeutic interventions. Mass spectrometry-based phosphoproteomics has generated an unprecedented resource of phosphorylation data across hundreds of human cell line studies. Systematic analysis of these datasets helps identify reliable and reproducible phosphorylation events, as well as co-regulation patterns, which reveal functional networks between proteins without requiring direct physical interaction or co-localization [21]. Previous studies using this framework have successfully mapped the phosphoregulatory landscapes of understudied kinases including CHEK1, BRAF, RAF1, and HIPK1 [22,23,24,25]. ROCK1 and ROCK2 are highly conserved paralogs that enable overlapping catalytic activities toward different substrates. However, extensive biochemical and genetic studies have demonstrated that the two isoforms exhibit non-redundant physiological functions, attributable to differences in tissue distribution, subcellular localization, regulatory interactions, and genetic knockout phenotypes rather than differences in their catalytic domains [1,7,8]. Unlike prior studies that characterized ROCK phosphorylation in single contexts or focused on direct substrates, we aimed to identify the phosphosites on ROCK1 and ROCK2 that are most frequently and reproducibly detected across diverse human cell line contexts and to determine whether positionally conserved phosphosites are associated with shared or paralog-specific co-regulatory network providing the first systems-level phosphoregulatory map of these kinases. To address these questions, we performed a systematic multi-criteria phosphoproteomic co-regulation analysis by integrating 3825 publicly available datasets associated with human cell line studies. We hypothesized that the most frequently phosphorylated sites on ROCK1 and ROCK2 would localize outside the kinase domain and, despite positional conservation, would involve paralog-specific co-regulatory networks reflecting their distinct physiological roles. Here, we applied an established multi-criteria phosphoproteomic co-regulation framework to ROCK1 and ROCK2, generating the first systems-level map of their predominant C-terminal phosphosites and associated regulatory networks, with implications for cytoskeletal and oncogenic signaling.

## 2. Results

### 2.1. Phosphoproteomic Dataset Compilation: Phosphosite Analysis of ROCK1 and ROCK2 and Systematic Identification of Predominant Phosphosites

**Figure 1:**
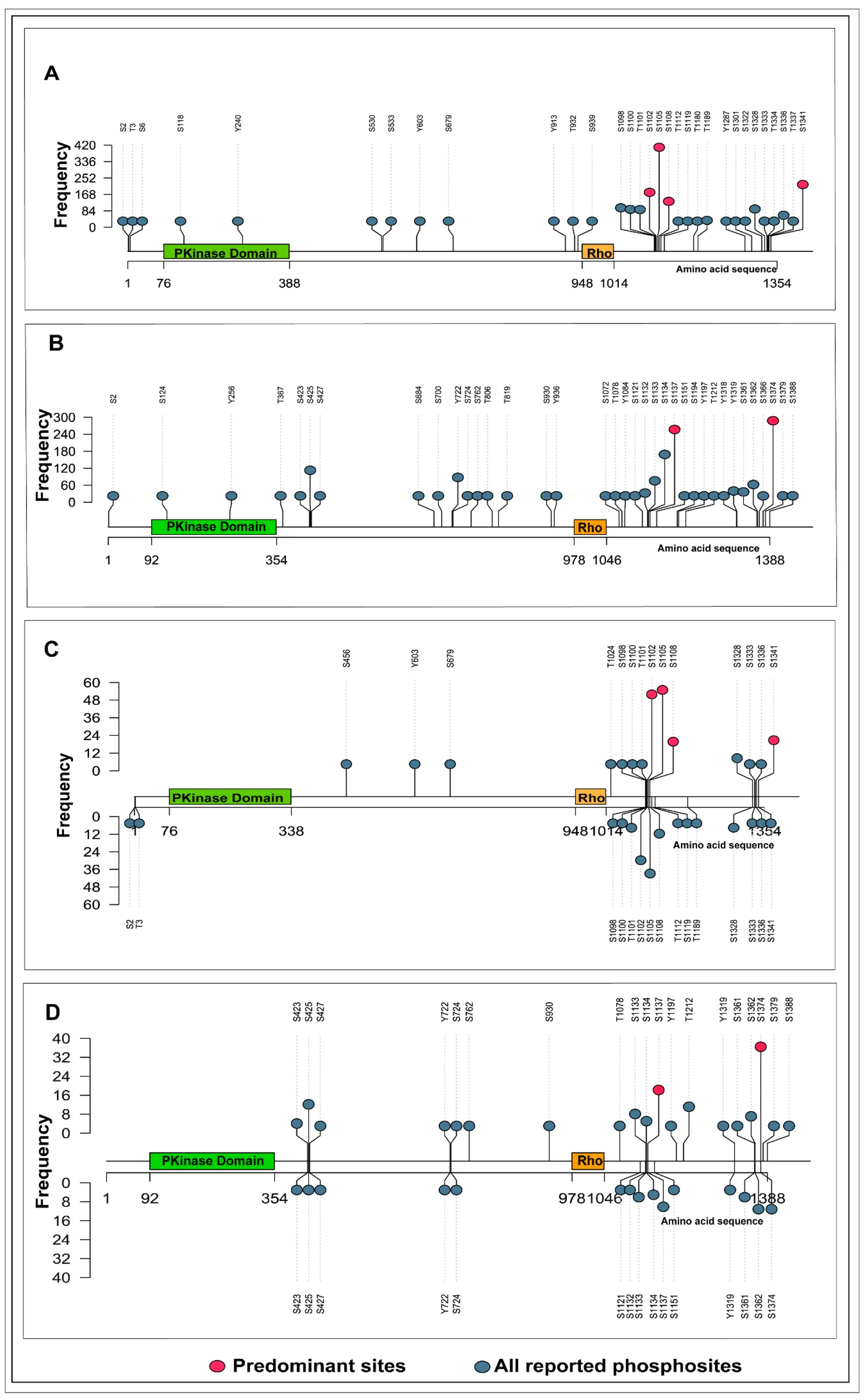
Lollipop plots illustrating the occurrence of phosphosites for ROCK1 and ROCK2 in human cellular profiling and differential phosphoproteomics datasets. The *x*-axis represents the amino acid sequence length, and the *y*-axis indicates the number of datasets in which each Class-1 phosphosite was detected. (**A**,**B**): Phosphosites of ROCK1 and ROCK2 identified in human cellular profiling datasets. (**C**,**D**): Phosphosites of ROCK1 and ROCK2 identified in human cellular differential datasets. The predominant phosphosites for both kinases are indicated in red.

A total of 3825 publicly available phosphoproteomic research articles from human cell lines were assembled, involving a wide range of biological and experimental conditions. These datasets represent individual biological or experimental conditions reported in published studies. Notably, multiple datasets were derived from single studies when independent conditions were analyzed separately. ROCK1 phosphosites were identified in 607 profiling datasets and 153 differential datasets, whereas ROCK2 phosphosites were present in 667 profiling datasets and 143 differential datasets (Supplementary Tables S1–S4). Across the profiling datasets, 31 distinct Class I phosphosites were detected on ROCK1, of which 19 were reported to be differentially regulated under at least one experimental condition. For ROCK2, 36 Class I phosphosites were identified in the profiling datasets, with 22 exhibiting differential regulation in at least one context. Cross-study heterogeneity arising from differences in phosphopeptide enrichment platforms, such as immobilized metal affinity chromatography (IMAC), titanium dioxide (TiO_2_), sequential enrichment of phosphopeptides (SIMAC), mass spectrometry instrumentation, and data-processing pipelines, was systematically addressed through uniform inclusion criteria: restriction to Class I phosphosites (localization probability ≥ 75% or A-score > 13), standardization of protein identifiers to HGNC gene symbols, harmonization of phosphosite annotations to UniProt canonical sequence accession numbers, and frequency-based thresholds requiring independent detection in at least three studies. Dataset integration was performed at the level of reported phosphosite identification and differential regulation as annotated within each individual study; raw mass spectrometry file reprocessing was not performed given the inconsistent availability of primary data files across literature. For ROCK1, the most frequent phosphosites were S1102 (detected in 189 profiling and 84 differential datasets), S1105 (419 profiling and 96 differential datasets), S1108 (143 profiling and 34 differential datasets), and S1341 (229 profiling and 26 differential datasets). For ROCK2, S1137 was identified in 264 profiling and 30 differential datasets, whereas S1374 was identified in 295 profiling and 49 differential datasets (Figure 1). Based on these detection frequencies, S1105 and S1341 were designated as the predominant phosphosites for ROCK1, and S1137 and S1374 for ROCK2.

### 2.2. Predominant Phosphosites Are Located Outside the Kinase Domain and Display Positional Conservation Between Paralogs

**Figure 2:**
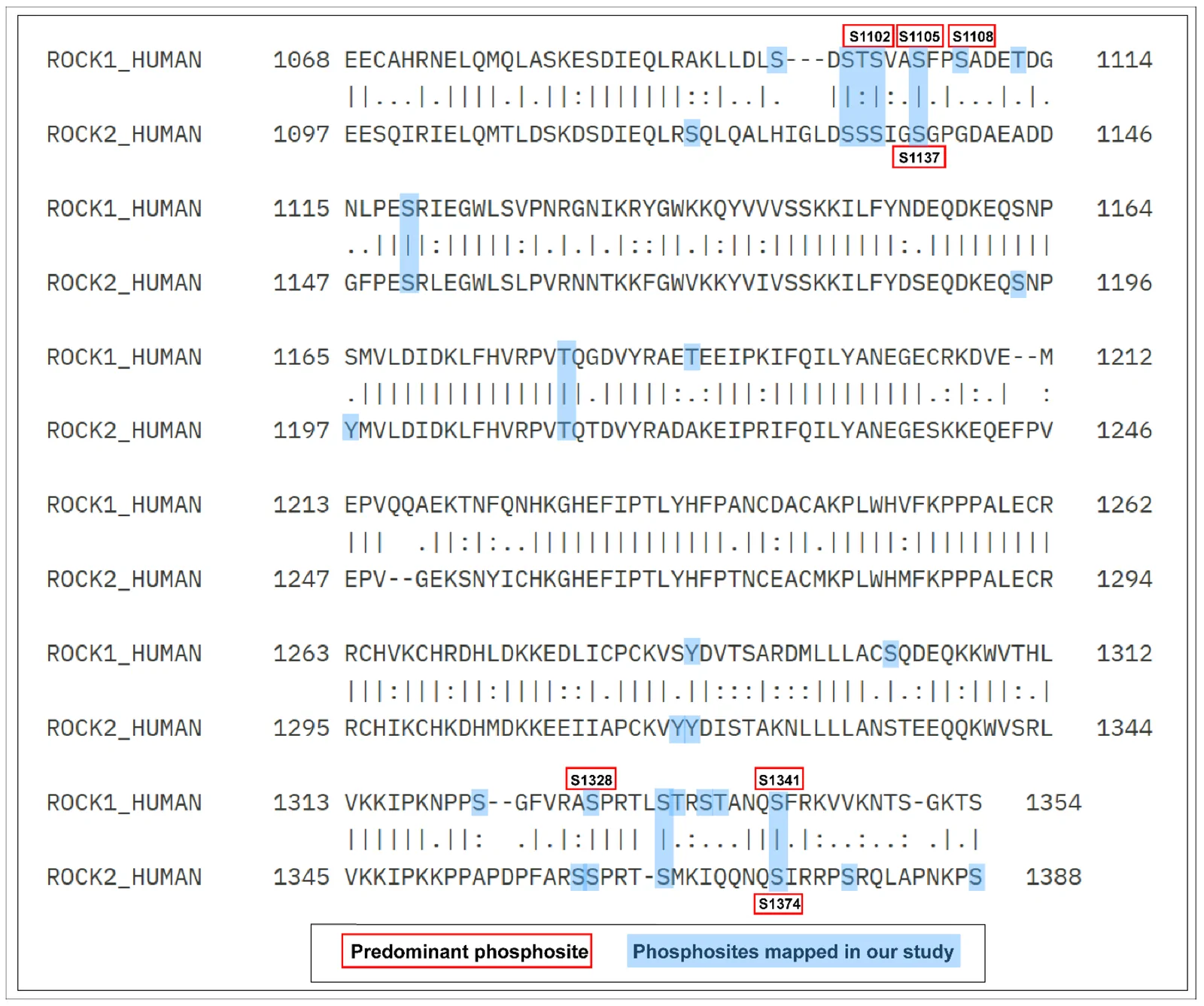
Partial sequence alignment depicting the predominant phosphosites of ROCK1 and ROCK2. The alignment spans residues 1068–1354 in ROCK1 and 1097–1388 in ROCK2. The phosphosites identified in this study are highlighted in blue, and predominant phosphosites are outlined in red.

Assessment of the ROCK1 tryptic peptide landscape revealed that S1102, S1105, and S1108 co-localized within a single phosphopeptide, introducing the possibility of co-detection bias. To minimize analytical redundancy, we selected a single representative phosphosite from this cluster based on its detection under independent experimental conditions. S1105 demonstrated the highest frequency of independent reporting across distinct studies (Supplementary Figure S1) and was therefore designated as the representative predominant phosphosite for this peptide cluster in all the subsequent analyses. Mapping the predominant phosphosites onto the domain architecture of ROCK1 and ROCK2 revealed that all four residues, S1105 and S1341 in ROCK1 and S1137 and S1374 in ROCK2, were localized in the C-terminal region distal to the kinase domain boundary. This positional characteristic classifies ROCK1 and ROCK2 as kinases with predominant phosphosites outside the kinase domain (POKD) [26]. To assess the degree of sequence conservation surrounding the predominant phosphosites between paralogs, we performed pairwise alignment of the canonical sequences of ROCK1 and ROCK2 using the EMBOSS NEEDLE algorithm [27]. The alignment spanning residues 1068–1354 of ROCK1 and 1097–1388 of ROCK2 revealed two pairs of positionally equivalent phosphosites: S1105 (ROCK1) and S1137 (ROCK2) occupy equivalent positions within the proximal C-terminal coiled-coil region, whereas S1341 (ROCK1) and S1374 (ROCK2) are positionally equivalent within the distal C-terminus (Figure 2). The sequence context immediately flanking each conserved pair was also found to be substantially conserved, indicating that these sites may be regulated by shared upstream kinases or serve equivalent regulatory functions within their respective paralogs.

### 2.3. Co-Regulatory Phosphosite Networks of Predominant ROCK1 Phosphosites

#### 2.3.1. ROCK1_S1105

Applying the multi-criteria FET framework, ROCK1_S1105 was found to be positively co-regulated with 141 high-confidence PsOPs across independent datasets (Supplementary Table S5). Proteins co-regulated with ROCK1_S1105 are annotated to functions including cardiomyocyte survival signaling, mitochondrial metabolic adaptation, genomic stability, and cytoskeletal homeostasis, suggesting potential functional coordination. Notably, PDPK1_S241, a master activator of the AKT/mTOR axis, was among the positively co-regulated PsOPs, which may reflect functional coordination, as PDPK1 and ROCK1 have been reported to physically interact at the plasma membrane in contexts involving RhoE displacement and actomyosin contraction. Additional co-regulated PsOPs include RPTOR_S877 and FOXO3_S7, implicating ROCK1_S1105 in negative feedback on the mTORC1 anabolic output and tumor-suppressive PI3K/FOXO3 crosstalk, respectively. Ten PsOPs were negatively co-regulated by ROCK1_S1105 (Supplementary Table S6).

#### 2.3.2. ROCK1_S1341

ROCK1_S1341 exhibited positive co-regulation with 204 PsOPs and negative co-regulation with 288 PsOPs (Supplementary Tables S7 and S8). The positive co-regulatory network includes BAD_S134, whose AKT-mediated phosphorylation enables 14-3-3-dependent mitochondrial sequestration and apoptosis suppression; WDR26_S121; PRKCA_S226, which converges on PI3K/AKT activation; and SOS1_S1161, a phosphorylation event established by RSK downstream of ERK that creates 14-3-3 docking sites and attenuates RAS/ERK amplitude via negative feedback loop. Co-regulation by DVL3_S48 and PRKCA_S226 positions ROCK1_S1341 at the juncture between non-canonical Wnt/PCP signaling (RhoA → ROCK1 → actin) and canonical Wnt/β-catenin signaling. The relatively large number of negatively co-regulated PsOPs (288) suggests that ROCK1_S1341 upregulation is associated with the suppression of opposing regulatory programs.

### 2.4. Co-Regulatory Phosphosite Networks of Predominant ROCK2 Phosphosites

#### 2.4.1. ROCK2_S1137

ROCK2_S1137 showed positive co-regulation with 610 PsOPs and negative co-regulation with 20 PsOPs (Supplementary Tables S9 and S10). The substantially larger positive co-regulatory network of ROCK2_S1137 compared to the cognate ROCK1 site (S1105; 141 PsOPs) indicates that, despite sharing a conserved sequence position, the two phosphosites are involved in markedly different cellular programs. The ROCK2_S1137 network is enriched in proteins involved in endosomal PI3K signaling dynamics, including PIK3C2A_S338, and AKT phosphorylation via spatially restricted class II PI3K activity. Co-regulation with AKT1_S121 is consistent with a feed-forward circuit in which ROCK2-mediated matrix stiffening drives FAK-dependent AKT1 activation, propagating signals to GSK3β and mTORC1 to promote cell survival and proliferation.

#### 2.4.2. ROCK2_S1374

ROCK2_S1374 exhibited the broadest co-regulatory network among all four predominant phosphosites, with 1349 positively and 73 negatively co-regulated PsOPs (Supplementary Tables S11 and S12). The network is enriched with proteins governing cytoskeletal remodeling, cell cycle progression, hypertrophic gene programs, profibrotic signaling, and maladaptive stress responses, consistent with the known pathophysiological roles of ROCK2 in cardiac hypertrophy, pulmonary fibrosis, and aggressive cancer phenotypes. Specific co-regulated PsOPs include MYC_S77 (stabilized by RAF-ERK signaling, increasing oncogenic transcriptional output), TP53_S315 (a bidirectional MAPK crosstalk node), EGFR_S1064 (initiating PI3K-AKT-mTOR cascades), IRS1_S636 (driving epithelial–mesenchymal transition and metastasis), and APC (the canonical Wnt tumor suppressor), as well as mTOR complex components, including RPTOR, RICTOR, RRAGC, and TSC2. Co-regulation with BCL9, a nuclear β-catenin co-activator that potentiates Wnt target gene transcription, further positions ROCK2_S1374 as a coordinator of transcriptional Wnt amplification in cancer cells.

### 2.5. Conserved Phosphosites Engage Overlapping but Predominantly Paralog-Specific Co-Regulatory Networks

A comparison of the co-regulatory networks of the two conserved phosphosite pairs demonstrated a consistent degree of paralog specificity, despite positional conservation. Of the PsOPs positively co-regulated with S1105 (ROCK1) and S1137 (ROCK2), only 19 proteins were shared, whereas 91 and 336 were uniquely associated with ROCK1 and ROCK2, respectively. Similarly, for the S1341/S1374 pair, the networks diverged substantially in terms of size and composition, with ROCK2_S1374 co-regulating a markedly diverse set of proteins. These data indicate that the conserved C-terminal phosphosites drive paralog-specific rather than redundant signaling programs in human cells, consistent with the known phenotypic non-redundancy observed in paralog-selective knock-out and pharmacological studies.

### 2.6. Upstream Kinases Co-Differentially Regulated with Predominant ROCK1 and ROCK2 Phosphosites

**Figure 3:**
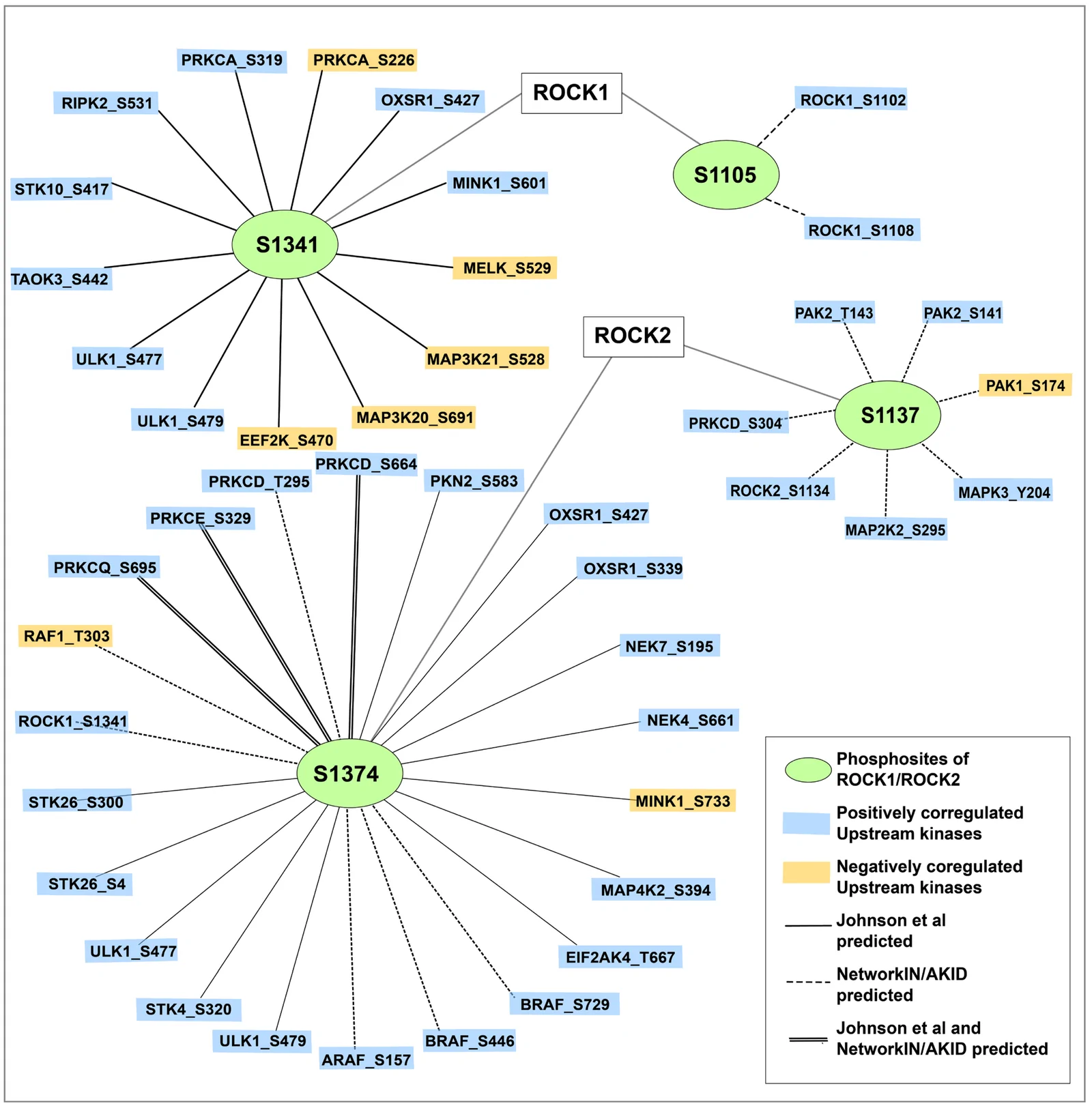
Co-regulatory Network Comparison. Representation of upstream kinases showing positive (blue) and negative (yellow) co-differential regulation with conserved phosphosites of ROCK1 (S1105 and S1341) and ROCK2 (S1137 and S1374).

Integration of co-regulation data with curated and predicted upstream kinase databases identified 15 kinases co-differentially regulated with ROCK1 predominant phosphosites and 26 with ROCK2 predominant phosphosites (Supplementary Tables S13 and S14). For ROCK1, 13 were predicted by kinase substrate profiling and two by NetworKIN/AKID. For ROCK2, 13 were from kinase profiling data and 13 from the NetworKIN/AKID databases. Several upstream kinases, including members of the CDK, MAPK, and DYRK families, were shared among the paralogs, consistent with ROCK regulation by both mitogenic and stress-responsive pathways. The larger upstream kinase network of ROCK2 (26 vs. 15 for ROCK1) further supports the broader regulatory engagement of ROCK2 compared to ROCK1 in diverse cellular states (Figure 3). ROCK2 negatively co-regulates RAF1_T303 with its predominant site S1374, consistent with the demonstrated physical interaction between the ERK-module phosphorylated form of RAF1 and ROKα (ROCK2), which inhibits ROCK2 activity, providing direct experimental support for the co-regulation network identified in this dataset [28].

### 2.7. Downstream Substrates Co-Differentially Regulated with Predominant ROCK1 and ROCK2 Phosphosites

**Figure 4:**
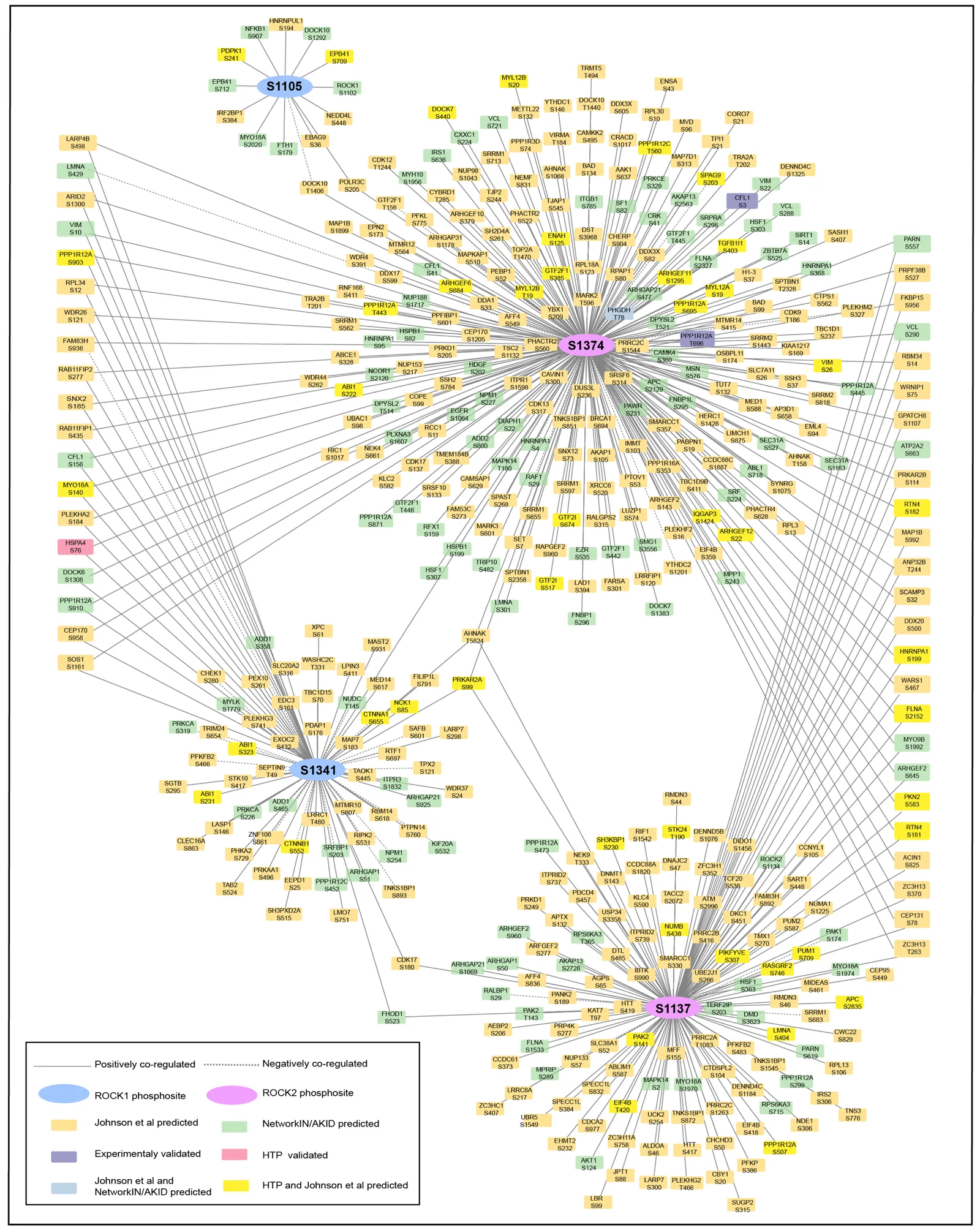
Downstream Substrate Co-regulation. Network representation of downstream substrates showing co-differential regulation with conserved predominant phosphosites of ROCK1 (S1341 and S1105) and ROCK2 (S1374 and S1137).

A total of 104 downstream substrates were co-differentially regulated with ROCK1 predominant phosphosites, of which 69 were predicted by kinase profiling, 34 by NetworKIN/AKID, and one by a high-throughput methodology (Supplementary Table S15). For ROCK2, 385 substrates were identified: 275 from kinase profiling, 106 from NetworKIN/AKID, one from high-throughput methods, and one that was experimentally validated (Supplementary Table S16). The expanded substrate network of ROCK2 aligns with its diverse physiological and pathological functions. This network facilitates the understanding that the C-terminal phosphosites of the paralog integrate various extracellular signals with cytoskeletal functions (Figure 4). Among the validated substrates, ROCK2 phosphorylates PPP1R12A (MYPT1) at T696, an interaction demonstrated using ROCK2 co-transfection and phospho-specific antibody detection (29) and CFL1 (cofilin) at S3, directly demonstrated in the context of dendritic spine polarity and PSD maturation (19).

### 2.8. Binary Interactors Co-Differentially Regulated with Predominant ROCK1 and ROCK2 Phosphosites

**Figure 5:**
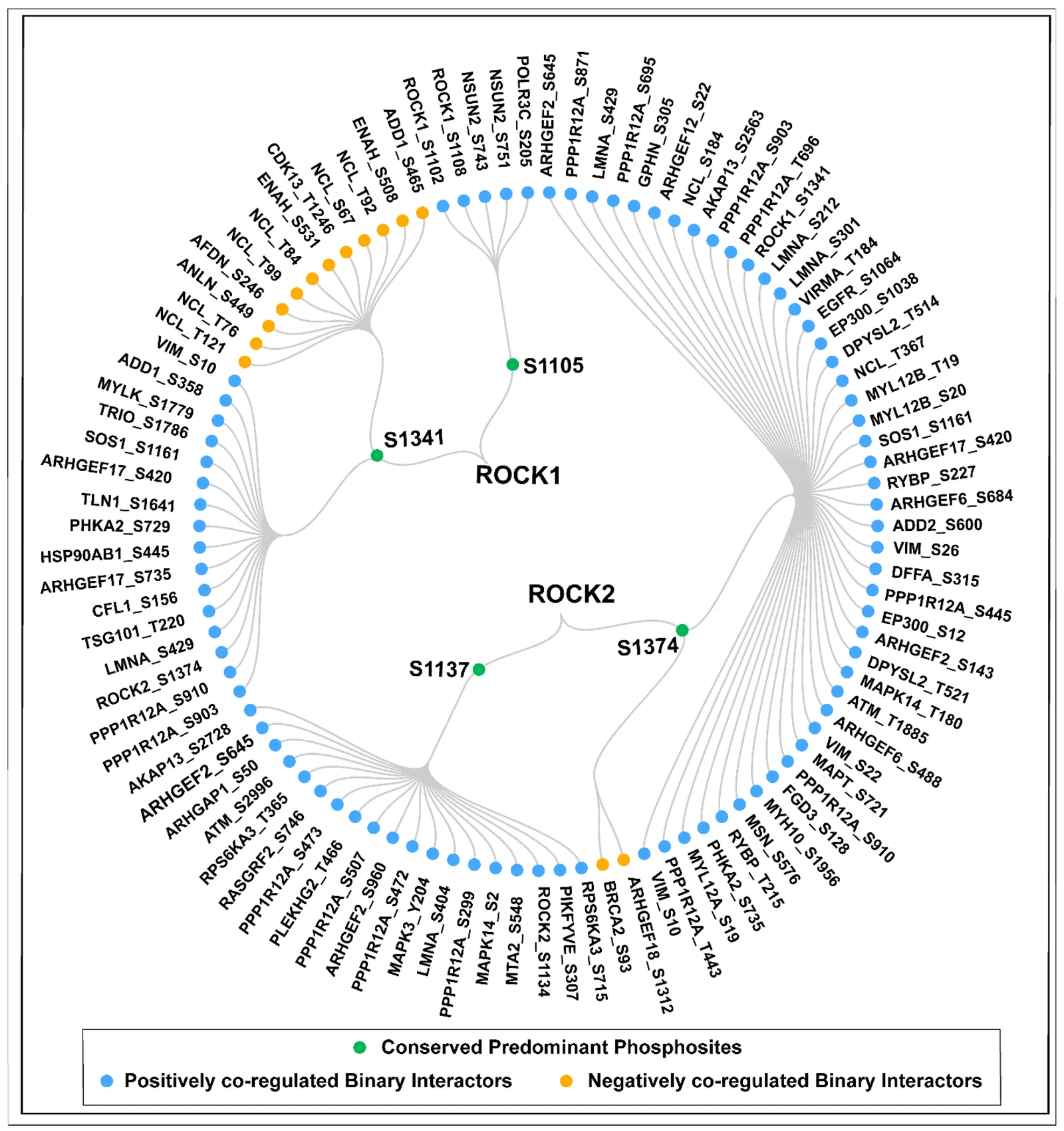
Circular dendrogram depicting predicted binary interactors of conserved predominant sites on ROCK1 and ROCK2.

Interactome curation identified 33 binary interactors of ROCK1 and 66 of ROCK2 from six protein–protein interaction databases (Supplementary Tables S17 and S18). Several interactors exhibited co-differential regulation with the predominant phosphosites of both paralogs, suggesting their participation in shared effector modules. These include MYPT1 (PPP1R12A), LIMK1, and MLC (MYL9), which are canonical ROCK substrates and interactors that are central to actomyosin contractility and cytoskeletal remodeling. Direct phosphorylation of the myosin regulatory light chain (MYL12A, Ser19) by Rho-kinase was among the earliest biochemical characterizations of ROCK activity [9], and this site is co-differentially regulated with ROCK2_S1374 in our dataset, further validating the interactome curation method. The broader interactome network of ROCK2 (66 interactors vs. 33 for ROCK1) is consistent with its wider tissue distribution in signaling-dense tissues, such as the brain and heart, and its more extensive co-regulatory phosphosite network. A circular dendrogram visualizing the predicted binary interactors and their co-regulation patterns is presented in Figure 5.

## 3. Discussion

### 3.1. The C-Terminal Phosphosites of ROCK1 and ROCK2 as Regulatory Hubs

The major observation of this study is that the most frequently phosphorylated sites on ROCK1 and ROCK2 in human cell line phosphoproteomics are not located within their kinase domains, but rather in the C-terminal coiled-coil region that encompasses the autoinhibitory PH domain. This is important because the C-terminus is the structural element through which GTP-RhoA relieves auto-inhibition [12,13]. We hypothesize that phosphorylation at or near the PH domain may modulate the threshold for kinase activation, C-terminal autoinhibitory affinity, or recruitment of regulatory proteins—a regulatory layer that would require experimental validation through kinase activity assays and domain interaction studies. The designation of ROCK1 and ROCK2 as POKD kinases [26] has functional implications, suggesting that the C-terminal phosphosites may serve as integration points for upstream signals that fine-tune ROCK activity, independently or in concert with the RhoA loading state.

The four predominant phosphosites, ROCK1_S1105, ROCK1_S1341, ROCK2_S1137, and ROCK2_S1374, occupy conserved sequence positions between the two paralogs. However, the co-regulatory phosphosite networks downstream of these conserved sites were largely non-overlapping, with fewer than 20 PsOPs shared between the S1105/S1137 pair, and the networks flanking S1341/S1374 similarly diverged. This dissociation between positional conservation and network divergence implies that the regulatory consequences of phosphorylation at these sites are context-sensitive and paralog-specific, consistent with the distinct tissue expression patterns, knockout phenotypes, and disease associations of ROCK1 and ROCK2.

### 3.2. Activation Sites of Predicted Upstream Kinases Reflect Paralog-Specific Signalling Outputs

Among the predicted upstream kinases, RAF1 phosphosites S29 and T303 were negatively co-regulated with ROCK2_S1374, consistent with the demonstrated physical interaction between the ERK-module-phosphorylated form of RAF1 and ROKα (ROCK2), which inhibits ROCK2 activity [28,29]. This inverse relationship suggests that ERK pathway activation and ROCK2 C-terminal phosphorylation are coordinated in an antagonistic manner, potentially reflecting the mechanism by which mitogenic ERK signaling restrains ROCK2-driven cytoskeletal contractility. For ROCK2, the activation sites of multiple predicted upstream kinases were positively co-regulated with its predominant phosphosites, reflecting a broader regulatory landscape for this paralog. These include BRAF_S446 and MAPK3_Y204 within the BRAF–MEK–ERK proliferative cascade, PAK2_S141 reflecting active Rac/Cdc42 signalling, and the activation sites of two PKC family members, PKCδ (PRKCD_T295) and PKCθ (PRKCQ_S695), implicating ROCK2 engagement during apoptotic and NF-κB-driven inflammatory signalling respectively [30,31,32]. The positive co-regulation of these kinase activation sites with ROCK2 predominant phosphosites, together with the downstream consequences of ROCK2 activity, connects ROCK2 to a broader oncogenic, apoptotic, and immune signalling landscape, consistent with the paralog divergence identified in this study.

### 3.3. ROCK1 and ROCK2 Phosphosite Co-Regulation in Oncogenic Signaling Axes

The co-regulatory phosphosite profiles of ROCK1 and ROCK2 converge on three major oncogenic axes: the RAS/RAF/MAPK pathway, the PI3K/AKT/mTOR cascade, and the Wnt signaling pathway. Within the MAPK axis, ROCK1_S1341 co-regulates SOS1_S1161, a phosphorylation event in which activated RSK establishes an ERK1/2 negative feedback loop via 14-3-3-mediated suppression of SOS1 [33]. This positions ROCK1 within a network that may coordinate with negative feedback on mitogenic RAS/ERK signaling; whether ROCK1_S1341 phosphorylation contributes to tumor suppression requires direct experimental testing. In contrast, ROCK2_S1374 co-regulates the pro-carcinogenic MYC_S77 phosphosite, which is stabilized through RAF-ERK-mediated reduction of GSK3β-dependent MYC degradation [34]. The juxtaposition of ROCK-MYC recapitulates the observation that MEIS1/2 loss simultaneously suppresses ROCK1 and elevates MYC, thereby influencing oncogenic proliferation [35]. Co-regulation with TP53_S315 suggests that ROCK2_S1374 is involved in bidirectional MAPK-p53 crosstalk. In this interaction, p38/JNK stabilizes p53 to promote apoptosis, whereas the absence of p53 allows sustained MAPK progression [36]. In addition, p53 upregulates miR-145 to directly inhibit ROCK1 [37], forming a tumor-suppressive p53-miR-145-ROCK1 axis.

Within the PI3K/AKT/mTOR axis, ROCK1_S1105 co-regulates PDPK1_S241, coupling PI3K signaling directly to cytoskeletal remodeling via ROCK1-mediated actomyosin contractility [38]. ROCK2 co-regulates AKT1_S121, establishing a feed-forward loop in which matrix stiffening drives FAK-dependent AKT1 activation and downstream mTORC1 engagement. The co-regulation of ROCK2_S1374 with EGFR_S1064, IRS1_S636, and core mTOR complex components (RPTOR, RICTOR, RRAGC, and TSC2) indicates that ROCK2 activity is broadly coupled to the PI3K/AKT/mTOR axis across multiple nodes, consistent with ROCK2’s established roles in resistance to PI3K inhibitors and mTOR-dependent hypertrophic gene programs [39,40].

In the Wnt pathway, ROCK1_S1341 co-regulation with DVL3_S48 positions ROCK1 as a cytoskeletal executor of non-canonical Wnt/PCP signaling: DVL3 activates RhoA upon Wnt ligand engagement, which activates ROCK1 to drive actin reorganization and direct cell motility [41,42]. ROCK2 co-regulates both APC and BCL9, which are components on opposite sides of the β-catenin destruction complex. This pattern suggests a state in which APC-mediated Wnt restraint is overcome, while ROCK2-driven motility is simultaneously enhanced, a functional uncoupling that may cooperate with APC loss in colorectal and other cancers to amplify both transcriptional Wnt activation and cytoskeletal invasion [43].

### 3.4. Paralog Divergence and Implications for Therapeutic Targeting

The markedly broader co-regulatory network of ROCK2 relative to ROCK1, particularly at S1374 (1349 PsOPs vs. 141 for ROCK1_S1105), is consistent with independent lines of evidence pointing to ROCK2 as the dominant paralog in cardiac hypertrophy, pulmonary fibrosis, and aggressive cancer conditions. [44]. The C-terminal region contains coiled-coil and pleckstrin homology (PH) domains that mediate intramolecular autoinhibition of the N-terminal kinase domain. The binding of active RhoA, RhoB, or RhoC to the Rho-binding domain disrupts this autoinhibitory conformation and activates the kinase [12,13]. Several phosphorylation events have been mapped to the C-terminal regulatory region of ROCK proteins. These include experimentally validated sites such as ROCK2 Ser1374, which is phosphorylated by Polo-like kinase 1 to enhance ROCK2 activation, and the activity-dependent autophosphorylation site ROCK1 Ser1333, which serves as a marker of ROCK1 activation [11,45,46]. In addition, recurrent phosphoproteomic studies have consistently identified ROCK1 Ser1341 as a frequently phosphorylated C-terminal residue, suggesting its potential physiological significance, although its regulatory function has not yet been experimentally established [47]. The ongoing clinical development of ROCK2-selective inhibitors underscores the therapeutic relevance of paralog selection. Our phosphosite-centric co-regulatory analysis provides a complementary systems-level perspective: whereas ROCK1 phosphosites are co-regulated with networks enriched for DNA damage responses and cardiomyocyte survival programs, ROCK2 phosphosites connect to far broader cytoskeletal, fibrotic, hypertrophic, and transcriptional networks, differences that could help in patient stratification strategies in clinical trials of ROCK inhibitors. Notably, 27 proteins shared between the S1105 (ROCK1) and S1137 (ROCK2) co-regulatory networks may constitute a core effector module common to both paralogs, potentially encoding the shared cellular functions of ROCK1/2, such as basic actomyosin contractility and tight junction integrity. Proteins unique to each network are candidate mediators of paralog-specific biology and are priority targets for experimental validation.

### 3.5. Limitations

In this study, the co-regulation framework was correlational by design and did not establish causal or mechanistic relationships between the phosphosites. The observation that ROCK1_S1105 and PDPK1_S241 are co-regulated across multiple datasets is consistent with functional annotation but does not demonstrate direct enzymatic relationships or define the order of events without experimental validation of the results. Variations in protein abundance can complicate the analysis of phosphosite co-regulation. As consistent protein-level quantification data were not available across all compiled datasets, this limitation could not be systematically addressed. We cannot rule out the possibility that some co-regulation patterns reflect coordinated changes in protein expression rather than phosphorylation-specific regulation. Future studies incorporating matched proteomic and phosphoproteomic data should clarify this distinction. The use of processed Supplementary Data instead of raw mass spectrometry files, combined with cross-study differences in phosphopeptide enrichment platforms (IMAC, TiO2, SIMAC) and mass spectrometry instrumentation, introduces variability in phosphosite localization and quantification that is only partially mitigated by strict Class I filtering and frequency-based thresholds. The functional significance of the predominant phosphosites ROCK1_S1105 and S1341 and ROCK2_S1137 and S1374 has not been validated using phospho-specific antibody approaches, site-directed mutagenesis, or direct kinase activity assays. However, the biological consequences of phosphorylation at these sites remain to be validated experimentally. Future studies should focus on conducting time-resolved phosphoproteomic experiments with paralog-selective ROCK inhibitors to validate the ROCK kinase activity and co-regulated PsOP networks identified in this study. Further studies should also focus on site-directed mutagenesis (Ser→Ala and Ser→Asp) at ROCK1_S1105, S1341, ROCK2_S1137, and S1374 to understand the functional implications of phosphorylation by developing and validating phospho-specific antibodies for the four predominant sites and incorporating protein abundance data to evaluate phosphorylation-specific and expression-driven co-regulatory patterns. In conclusion, this analysis aggregates phosphoproteomic data across diverse cell types, experimental conditions, and disease contexts without stratification. Although this broad integration increases statistical power, it may mask context-specific co-regulation patterns, such as those unique to cardiac hypertrophy, fibrosis, or specific cancer subtypes. Future studies should stratify datasets by biological context to resolve condition-dependent regulatory networks.

## 4. Materials and Methods

### 4.1. Assembly of Human Cell-Line Phosphoproteomic Datasets

A comprehensive collection of 3825 publicly accessible human cell line phosphoproteomic datasets was assembled from peer-reviewed literature identified through PubMed using the MeSH terms “phosphoproteomics” OR “phosphoproteome,” excluding plant studies and review articles. Each dataset corresponds to a specific biological or experimental condition, and a single publication may contribute to multiple datasets. Data were sourced from the Supplementary Tables of published studies and, wherever available, from public repositories, including PhosphoSitePlus and ProteomeXchange databases. Due to variability in raw data and availability across studies, no reprocessing of raw mass spectrometry files was performed; integration was conducted at the level of reported phosphosite identifications and differential regulation annotations as defined within each original study.

Datasets were classified into two categories: (i) profiling datasets, which are qualitative inventories that enumerate detected phosphosites under a given experimental condition, and (ii) differential datasets, which are quantitative comparisons that report phosphosites as upregulated or downregulated relative to the control condition. Differential expression thresholds were standardized as fold-change ≥1.3 for upregulation and ≤0.76 for downregulation, with *p* < 0.05. The phosphopeptide enrichment methods for each dataset, such as IMAC, TiO_2_, and SIMAC, were also documented.

To address inter-study heterogeneity arising from differences in enrichment methods, instrumentation platforms, and data processing pipelines, uniform inclusion criteria were applied: (i) restriction to Class I phosphosites (localization probability ≥ 75% or A-score > 13); (ii) standardization of all protein identifiers to HUGO Gene Nomenclature Committee (HGNC) gene symbols (downloaded 30 May 2023) [48]; and (iii) harmonization of individual phosphosite annotations to UniProt canonical accession numbers using an in-house mapping tool.

### 4.2. Identification of the Predominant Phosphosites in ROCK1 and ROCK2

Class I phosphosites of ROCK1 were extracted from the assembled datasets. The frequency of detection for each phosphosite was quantified separately across the profiling and differential datasets. Phosphosites were ranked by detection frequency; those most consistently observed across the greatest number of independent profiling and differential datasets were designated as predominant phosphosites. Phosphosites that did not meet the Class I criteria or were detected in fewer than three independent studies were excluded from the downstream analysis. Where multiple phosphosites were identified within the same tryptic phosphopeptide (creating the possibility of redundant co-detection), the most frequently detected and differentially regulated site was selected as the representative for subsequent co-regulation analysis to minimize analytical redundancy and ascertainment bias [24]. Phosphosite distribution was visualized using lollipop plots generated with the R/Bioconductor package trackViewer (v1.47.0).

### 4.3. Sequence Analysis of ROCK1 and ROCK2

To evaluate sequence similarity, the canonical protein sequences of ROCK1 (UniProt ID: Q13464) and ROCK2 (UniProt ID: O75116) were analyzed. To determine the degree of similarity between the two proteins, pairwise sequence homology was assessed using EMBOSS NEEDLE via the EMBL-EBI Job Dispatcher web service (https://www.ebi.ac.uk/jdispatcher/psa/emboss_needle/) [27]. To identify conserved domains and motifs across the ROCK1 and ROCK2 sequences, multiple sequence alignments were performed using Clustal Omega via the EMBL-EBI Job Dispatcher web service (https://www.ebi.ac.uk/jdispatcher/msa/clustalo/) [49].

### 4.4. Co-Regulation Analysis of Phosphosites in Other Proteins

For each predominant ROCK1 and ROCK2 phosphosite, phosphosites in other proteins (PsOPs) that were co-detected in the same differential datasets were systematically sorted. Co-regulation directionality was encoded using paired category codes: UU (both sites upregulated), DD (both downregulated), UD (ROCK site upregulated and PsOP downregulated), and DU (ROCK site downregulated and PsOP upregulated). Positive co-regulation includes UU and DD patterns (UUDD), whereas negative co-regulation includes UD and DU patterns (UDDU) [23,50,51]. The statistical significance of co-regulation was assessed using a one-sided Fisher’s exact test (FET) applied to a 2 × 2 contingency table constructed for each phosphosite pair.

Fisher’s Exact Test (FET):

$$\sum p=\frac{\left(a+b\right)!\left(c+d\right)!\left(a+c\right)!\left(b+d\right)!}{n!}\sum_{i}\frac{1}{a_{i}!b_{i}!c_{i}!d_{i}!}$$

In the contingency table, cell ‘a’ denotes conditions in which neither phosphosite in the pair was differentially detected; ‘b’ denotes conditions in which only one site was detected; ‘c’ denotes conditions showing opposite directional regulation (negative co-regulation); and ‘d’ denotes conditions showing same-direction co-regulation (positive co-regulation). High-confidence co-regulated PsOPs were identified by applying four successive filtering criteria: (i) detection frequency in the co-regulated category ≥10% of the total differential datasets containing the predominant phosphosite; (ii) FET *p*-value < 0.05; (iii) co-regulation reported across ≥3 independent publications (PMID confidence threshold); and (iv) observed in ≥3 distinct experimental conditions (condition code threshold). These criteria collectively define reproducible, high-confidence co-regulation patterns that are robust against dataset heterogeneity and experimental confounders.

### 4.5. Extraction of Upstream Kinases, Substrates, and Protein Interaction Partners of ROCK1 and ROCK2

The experimentally validated upstream kinases and protein substrates, along with their phosphosites, associated with ROCK1 and ROCK2 were curated from databases such as PhosphoSitePlus [52] (downloaded on 22 May 2023), Phospho.ELM 9.0 [53] (downloaded on 24 May 2023), and RegPhos 2.0 [54] (downloaded on 24 May 2023). Upstream kinases and substrates specific to ROCK1 and ROCK2 were predicted using multiple in silico prediction tools, including NetworKIN [55] (predicted on 4 January 2023), AKID [56] (predicted on 24 May 2023), and iKiP-DB (HTP) [57]. These predictions were assembled for the kinase-substrate relationship analysis. API support was used to process the entire RefSeq protein sequences because most prediction tools require individual protein sequences as input. Additionally, all substrates and kinases of ROCK1 and ROCK2 phosphosites derived from synthetic peptide screening by Johnson et al. (2023) [58] were incorporated (90th percentile cut-off). The protein–protein interactors of ROCK1 and ROCK2 were extracted from several databases such as HPRD [59], BIND [60], BioGRID [61], ConsensusPathDB version 35 [62] (downloaded on 22 May 2023), CORUM [63] (downloaded on 3 March 2023), and RegPhos 2.0 [54] (downloaded on 24 May 2023). All datasets were cross-referenced with the co-regulation results to identify kinases, substrates, and interactors showing significant co-differential regulation of ROCK1 and ROCK2 predominant phosphosites.

### 4.6. Data Visualization

Lollipop plots illustrating the phosphorylation patterns of ROCK1 and ROCK2 phosphosites were generated using the AR/Bioconductor package, trackViewer (v1.47.0) [64]. The phosphosite distribution across the qualitative profile datasets was visualized using the Python (v3.13.0) packages Matplotlib (v3.10.0) and Pandas (v2.3.3). Circular dendrograms representing the co-regulation between interactors and substrates were plotted using RAWGraph 2.0 [65]. Other data visualizations and figure editing were performed using Cytoscape (v3.10.4) [66] and Adobe Illustrator (2020), respectively.

## 5. Conclusions

This study presents a comprehensive phosphoproteomic analysis of ROCK1 and ROCK2, identifying paralog-specific co-regulatory networks associated with the predominant phosphorylation sites and generating hypotheses regarding their roles in the integration of cytoskeletal and oncogenic pathways. Through systematic mining of 3825 human cell line phosphoproteomic datasets, we predicted that the most frequently and reproducibly phosphorylated sites on ROCK1 and ROCK2 localized to conserved positions within the C-terminal region, outside the kinase domain. These sites, ROCK1_S1105, ROCK1_S1341, ROCK2_S1137, and ROCK2_S1374, are predicted to serve as co-regulatory nodes linking cytoskeletal signaling with oncogenic MAPK, PI3K/AKT/mTOR, and Wnt pathway programs, based on phosphosite co-occurrence patterns. Despite positional conservation, the co-regulatory networks of ROCK1 and ROCK2 are largely paralog-specific, with ROCK2 coordinating a substantially broader and more diverse phosphosite network.

## Data Availability

The original contributions presented in this study are detailed in the article and Supplementary Materials. Further inquiries may be directed to the corresponding authors. In preparing this article, the authors employed Grammarly software to correct basic grammatical errors. The authors have thoroughly reviewed and edited the output and take responsibility for the content of this publication.

## Supplementary Materials

The following supporting information can be downloaded at: https://www.mdpi.com/article/10.3390/ijms27188121/s1.
